# Changes in screen time activity in Norwegian children from 2001 to 2008: two cross sectional studies

**DOI:** 10.1186/1471-2458-13-80

**Published:** 2013-01-29

**Authors:** Nina C Øverby, Knut-Inge Klepp, Elling Bere

**Affiliations:** 1 Department of Public Health, Sport and Nutrition, Faculty of Health and Sport, University of Agder, Kristiansand, 4604, Norway; 2 Department of Nutrition, Faculty of Medicine, University of Oslo, Oslo, 0316, Norway

**Keywords:** Screen time, Children, Norway

## Abstract

**Background:**

There has been an increase in screen-based communication, leading to concerns about the negative health effects of screen-based activities in children and adolescents. The present study aimed to (1) analyze changes in screen time activity in Norwegian children from 2001 to 2008, and (2) to analyze associations between the changes in screen time activity over time and sex, grade level and parental educational level.

**Methods:**

Within the project Fruits and Vegetables Make the Marks (FVMM), 1488 6^th^ and 7^th^ grade pupils from 27 Norwegian elementary schools completed a questionnaire including a question about time spent on television viewing and personal computer use in 2001 and 1339 pupils from the same schools completed the same questionnaire in 2008. Data were analyzed by multilevel linear mixed models.

**Results:**

The proportions of 6^th^ and 7^th^ grade pupils at the 27 schools that reported screen time activity outside school of 2 hours/day or more decreased from 55% to 45% (p<0.001) from 2001 to 2008 when adjusting for sex, grade level and parental education. The decrease was most evident in 6^th^ graders (51% to 37%) and in children with highly educated parents (54% to 39%).

**Conclusion:**

The present study shows that there has been a marked reduction in screen time activity outside school in this group of Norwegian 10–12 year olds from 2001 to 2008.

## Background

Over the past 20 years there has been an expansion in screen-based communication and entertainment available to adolescents [1]. Following this, there has been an emerging concern about the negative health effects of screen time activity in children and adolescents. These effects include less time for physical activity [2], poorer academic performance [3], aggressive behaviour [4], higher energy-intake [5], more physical complaints [6], higher risk of overweight [7,8] and other metabolic risk factors [9]. In addition, a recent review concluded that sedentary behaviours track at moderate levels from childhood to adolescence [10]. The WHO Global Strategy on Diet, Physical Activity and Health identified physical inactivity together with poor nutrition as major risk factors for some of the leading causes of mortality [11].

A systematic review on correlates of screen-viewing show that screen-viewing is not equally distributed across social and demographic groups [12]. A Norwegian study found differences in screen time activity between the sexes [13], that screen time increase with age [13], and that higher parental education is related to less time spent on screen activities [13]. Results from a recent study among Norwegian adolescents showed that more screen time increased the risk of overweight and obesity [13] and there was also a clear link between overweight and social class; the prevalence of overweight and obesity was higher in children of parents with lower education [13]. In Norway, the prevalence of overweight among 11-year olds is 14–16% [13-15] and the prevalence of obesity is about 1–2% [13-15], which is lower than for other European countries, according to results from the ENERGY-project [15]. The prevalence of obesity is increasing with age and about 20% of Norwegian adults are classified as obese [16]. Among obese ethnic Norwegian adolescents, the prevalence of metabolic syndrome is 30% [17]. Compared with other European countries, like Hungary and Greece, the Norwegian adolescents have a more favourable cardiometabolic risk profile [18]. There are no studies presenting secular trends of overweight for 11-year-olds in Norway; however, data on 9-year-olds show an increase of overweight children from 2008 to 2010 in Norway [19]. With the increasing number of overweight children, supervising risk factors like sedentary behaviour is important.

In 2001 The American Academy of Pediatrics recommended that paediatricians should recommend that parents limit children’s total media time to no more than 2 hours a day [20]. This recommendation has been implemented in national recommendations in Australia [21] and in the USA [20]. Although there are no such recommendations in Norway, there have been some initiatives to increase physical activity in children and adolescents [22]. Since 2001 the Directorate of Health has started surveillance of activity and inactivity in national studies. They have implemented an action plan on physical activity [23]. This action plan focused on active leisure time, active everyday life and active environment by encouraging the experience of outdoor life, securing areas for public sojourn and travel, and promoting opportunities for physical activity in kindergartens, schools and at work. The action plan did not specifically focus on reducing screen time; however, by increasing active leisure time, a reduction in screen time might be observed. A study among Norwegian children found clear relations between physical activity (PA) and sedentary behaviour, where high PA was correlated with less screen time [2]. However, some researchers have found that PA and sedentary behaviour represent different paradigms [24] and that preventive action against metabolic risk in children may need to target TV viewing and PA separately [25].

Few studies have assessed the longitudinal pattern of screen time activity [26-29]. A recent American study showed that levels of screen time activity were stable in a nationally representative sample of children aged 9–13 year olds from 2001–2006 [26]. An Australian study found that from 1997 to 2001 there was a decrease in number of children who met the guideline of less than 2 hours of screen time activity [27]. Results from the *Health Behaviour in Schoolaged Children* (HBSC) study showed that there was an increase in hours spent on personal computers (PC) per week in Norwegian 6^th^, 8^th^ and 10^th^ grades from 1997 to 2005 [30]. Further results showed that there was a decrease in time spent in front of TV from 1997 to 2005 [30]. No study has assessed time changes in screen time activity in Norwegian children and adolescents in the same region after 2005. Cross sectional studies have shown that 36-42% of Norwegian 6–8 year olds and 56-65% of Norwegian 9–11 year-olds [13] exceed the 2 hour screen time per day recommendation. This is line with other countries where many children do not meet the recommendations [31]. Recent results from the ENERGY-project show that Norwegian boys and girls spend 168–196 minutes per day in front of screens [15].

The aim of this study was (1) to analyze changes in screen time activity in Norwegian children from 2001 to 2008, and (2) to analyze associations between the changes in screen time activity over time and sex, grade level and parental educational level.

## Methods

### Sample and procedure

This study is a part of the Fruits and Vegetables Make the Marks (FVMM) project. In 2001, 48 schools from Hedmark and Telemark counties (24 schools in each county) were randomly selected and invited to participate in the project FVMM. In total, 38 schools agreed to participate in the study. All sixth and seventh graders (age 10–12 years) in these 38 schools were invited to take part in a questionnaire survey. These 38 schools were contacted again in 2008, and were invited to participate once more in a similar survey. At that time 27 schools agreed to participate, and all sixth and seventh graders in these 27 schools were invited to participate. Pupils from these 27 schools in 2001 and in 2008 constitute the study samples of the present study.

During both data collections (in 2001 and 2008), the same questionnaire was completed by the children in the presence of a trained project worker in the classroom. One school lesson (45 min) was used to complete the questionnaire, and participating children brought home a parent questionnaire to be completed by one of the parents. Written informed consent was obtained from parents and children prior to participation in the study. Research clearance was obtained from The Norwegian Social Science Data Services.

The present study sample includes in total 2827 children (1488 in 2001 and 1339 children in 2008) out of 3439 eligible (participation rate 82.2%). The main reason why children did not participate in the study was absence from school on the scheduled day for the survey. The parent questionnaire was completed by a parent of 83% and 74% of the participating children in 2001 and in 2008, respectively. Descriptive statistics for the 2001 and 2008 samples are presented in Table 1. Further details on sampling process and the study sample are presented elsewhere [32].

**Table 1:** Description of participants included in 2001 and 2008

	**2001**	**2008**	p-value*
Number of schools	27	27	
Eligible pupils	1727	1712	
**Participating pupils**	1488	1339	
Participation rate pupils (%)	86%	78%	
Sex pupils (% girls)	50%	52%	0.21
Age pupils (% 7^th^ graders)	47%	49%	0.50
TV/PC watching (% ≥2 hours/day)	55%	45%	<0.001
Mean time spent on TV/PC watching (hours/day) (mean (SD))	2.1 (1.5)	1.7 (1.4)	<0.001
*Time spent on screen time*, *pupils* (%):			
Spend no time on screen time activity	19 (1)	19 (1)	
Less than ½ hour/day	150 (10)	155 (12)	
½-1 hour/day	484 (33)	544 (41)	
2-3 hours/day	515 (35)	429 (33)	
4 hours/day	133 (9)	80 (6)	
More than 4 hours/day	160 (11)	90 (7)	<0.001
**Participating parents**	1230	996	
Participation rate parents (%)	83%	74%	
Sex parents (% women)	85%	78%	<0.001
Age parents (mean, years)	39.9	41.1	<0.001
Education, parents (% with higher education)	42%	54%	<0.001

*P values are based on t-tests for continuous variables and on chi-square tests for dichotomous variables.

### Instrument

The questionnaire included one question on screen time: *Excluding school hours*, *how many hours a day do you usually watch TV or use a computer*? Response alternatives were: *None*, *less than 0*.*5 hour*/*day*, *0*.*51 hour*/*day*, *23 hours*/*day*, *4 hours*/*day*, and *more than 4 hours*/*day*. This variable was dichotomized into less than 2 hours/day (=0) and 2 hours/day or more (=1). This was done according to the American Academy of Pediatrics recommendation on screen time. In addition, the response alternatives were recoded into hours/day (seldom=0, less than ½ hour/day=0.25, ½-1 hour/day=0.75, 2–3 hours/day=2.5, 4 hours=4, more than 4 hours=5). Mean time spent on screen activities was calculated. This question originates from the HBSC-study [6]; however, the question was changed to include both TV and PC use in the present study, while TV and PC use were separate questions in the HBSC-study. Based on data from a previous test–retest study involving 114 children from 6th grade (fruit and vegetable reliability has been presented elsewhere [33]), the question regarding screen time activity yielded a Spearman coefficient of r=0.67, indicating acceptable reliability on two assessments 14 days apart.

The children were asked to identify their gender. Parental education level was assessed individually in the questionnaire filled in by the parents, using the question: “*What level of education do you have*?”. The question had four response alternatives: “*elementary school*”, “*high school*”, “*college or university*” (3 years or less) and “*college or university*” (more than 3 years). This variable was dichotomized (lower: no college or university education vs. higher: having attended college or university).

### Statistical analysis

In Table 1, differences from 2001 to 2008 in sex, parental education, screen time behavior and physical activity were analyzed by chi-square and t-test statistics.

The main analysis was a multilevel linear mixed model with the dichotomized screen time variable as the dependent variable. According to the literature, there might be a difference in screen time activity depending on sex, age, and parental education. Therefore, the model included time of data collection, sex, parental education and grade level, as well as the following interactions terms: time*sex, time*parental education and time*grade level to investigate changes over time. All models were also adjusted for school as a random effect. Crude models of time trends according to sex, grade level and parental education are also presented in Table 2.

**Table 2:** Crude and adjusted proportion (%) of children reporting a screen time activity of 2 hours/day or more according to sex, class, and parental education stratified by the year the study was performed*

	**CRUDE**						ADJUSTED*					
	**2001**		**2008**		**Change (percentage points)**	**p**	**2001**		**2008**		**Change (percentage points)**	**P for time x group interaction**
	**% (95% CI)**		**% (95% CI)**				**% (95% CI)**		**% (95% CI)**			
	**(n=1461)**		**(n=1317)**				**(n=1209)**		**(n=979)**			
All	55	(53, 58)	45	(43, 48)	−10	0.000	55	(52, 59)	45	(42, 49)	−10	
***Sex***												0.661
Boys	61	(58, 65)	51	(47, 55)	−11	0.000	60	(55, 64)	51	(46, 55)	−9	
Girls	49	(46, 53)	41	(37, 44)	−9	0.001	51	(47, 55)	40	(35, 44)	−11	
***Grade level***												0.022
6	51	(48, 55)	38	(34, 41)	−13	0.000	51	(47, 56)	37	(32, 41)	−15	
7	60	(56, 63)	54	(50, 58)	−6	0.023	59	(55, 64)	54	(49, 59)	−5	
***Parental education level***												0.044
Low	57	(53, 60)	51	(47, 56)	−5	0.079	57	(53, 61)	51	(46, 56)	−6	
High	53	(49, 58)	39	(34, 43)	−15	0.000	54	(49, 58)	39	(35, 44)	−14	

*Multilevel linear mixed models adjusted for all variables presented in the table and school as a random effect.

A school attrition analysis has been conducted and reported previously [34], where pupils at the 27 schools in the study sample were compared with those at the 11 schools participating in 2001 but not in 2008, regarding sex, parental educational level, and unhealthy snacks at school and all day. A t-test was used for continuous variables and chi-square statistics for the categorical variables. No significant differences between the study sample and pupils at schools not participating in 2008 were observed.

Accepted significance level was set at p<0.05. All statistical analyses were performed using the SPSS statistical software package version 17.0 (SPSS Inc. Chicago, IL, USA).

## Results

Unadjusted, the proportion of 6^th^ and 7^th^ grade pupils at the 27 schools that reported screen time activity outside school of 2 hours/day or more decreased from 55% to 45% (p<0.001) from 2001 to 2008 (Table 1). Mean reported screen time activity was 2.0 hours/day in 2001 and 1.7 hours/day in 2008 (Table 1). The parents were older in 2008 than in 2001 (41.1 years vs 39.9 years, p<0.001) and a higher proportion were highly educated (54% vs 42%, p<0.001).

Exploring the differences in screen time activity further, Table 1 shows that in 2008 compared with 2001 a higher proportion of participants reported spending 0.5-1 hour a day (41% vs 33%) and a lower proportion reported to spend 2–3 hours/day on screen time activity (33% vs 35%).

Both crude and adjusted models are presented in Table 2. The crude model shows a decrease in the proportion reporting screen time activity of 2 hours/day or more for all groups, except for those with parents with low education. In the fully adjusted model the proportions reporting screen time activity of 2 hours/day or more were also 55% (95% CI: 52–59) in 2001 and 45% (95% CI: 42–49) in 2008 (Table 2). Significant interactions were observed for time*grade level (p=0.02), time*parental education (p=0.04), but not for time*gender (p=0.66). From 2001 to 2008 the proportion of 6^th^ graders with a high level of screen time activity decreased more than the proportion of 7^th^ graders, by 15 and 5 percentage points, respectively (Table 2). Similarly, a greater decrease in the proportion of children with a high level of screen time activity was observed among those with parents with higher education, compared with those with parents without higher education, with decreases of 14 and 6 percentage points, respectively. More boys than girls engaged in 2 hours/day or more of screen time activities at both points in time, but there were no difference over time, with decreases of 9 and 11 percentage points, respectively, from 2001 to 2008.

## Discussion

There has been a marked reduction in screen time activity outside school in 10–12 year olds from 2001 to 2008 in the present study sample. The decrease is most evident in 6^th^ graders and in children whose parents have a high educational level.

Few studies have described time change in screen time activity in children during the past decade. Some previous studies have reported an increase in the number of children exceeding the recommended screen time per day [28,29,35], while a recent American study found that time spent on screen time activities was stable from 2001 to 2006 among children and adolescents and that more than 75% met the guidelines of not exceeding 2 hours a day [26]. In Norway, the HBSC-study showed an increase in time spent on personal computers and a decrease in time spent watching TV in adolescents from 1997 to 2005. The reduction found in total screen time activity in the present study is surprising; thus, exploring the limitations with the question posed is warranted. In our study, participants were asked to report total time spent on all screen time activity. From 2001 to 2008, there has been an increase in computer usage in Norway, e.g., the proportion having a PC at home has increased from 75% to 90% in the general Norwegian population [36]. This change might have led the participants to interpret the question differently in 2008 compared with 2001. The HBSC-study changed the question used in 2005 because of new screen activity use and divided the question regarding PC use into the two categories of a) TV-games, PC-games and b) chatting and surfing. Having such a wide question as in the present study might have led to underreporting of newer screen time activities in 2008 compared with 2001, such as homework, chatting, internet gaming, use of cell phones, etc. The general reduction found in the present study might also in part be a result of the already mentioned initiatives from health authorities on increasing physical activity to limit sedentary behavior. National studies in adults show an increased knowledge regarding the negative effects of too much screen time activity and the benefits of increased physical activity [22], supporting the possibility that these initiatives might have had a positive impact. In addition, the observed differences in parental age and education between 2001 and 2008 might also partly explain the decrease, as older parents may be more traditional in their approaches to child rearing and may let their children spend less time on video games and computers [37].

In line with the above discussion, it is interesting to note that the observed changes in screen time activity were more evident among specific socio-demographic groups. There was a marked reduction in screen time activity among those with highly educated parents. This is consistent with the results of previous research [3,38-40], showing that parents with higher education are more likely to follow health recommendations and act on health authority initiatives than those without higher education. Several studies find that maternal educational level is consistently inversely associated with children’s television viewing [38].

Most studies find, like the present one, that older children are more likely than younger ones to spend more than 2 hours daily on screen time activities [2,12,31,38]. The marked reduction among 6^th^ graders may reflect several aspects. First, the younger the children are, the more likely they are to be supervised and have strict rules regarding screen time activities [31]. Second, demands to use computers for home work are more frequent with increasing age, and older children tend to have greater access to media sources than do younger children [31].

The present results suggest that boys spend more time than girls on screen time activities. This is comparable to findings from other studies investigating sex differences in screen time activities [2,31]. However, studies including new sedentary behaviors, such as talking on the phone, texting, and instant messaging, find that girls are more likely to report more time spent on these activities than boys [41]. Other studies have also found that boys report being engaged in more physical activity compared with girls [42], suggesting that girls spend their leisure time doing other activities than physical activity and screen based activity. In the present study, we observed no differences in screen time activity between sexes over time.

Strengths and limitations: This study includes two cross-sectional surveys seven years apart in a well-defined population from two Norwegian counties. It included a high number of schools, and the participation rate for the children was high. The same question, with proven acceptable reliability in this age group, was used to measure screen time activity exposure at both time points.

However, it is a clear limitation of this study that the assessment tool used for screen time activity included one question only. Thus, we were not able to assess time spent on different activities such as TV-viewing, gaming, and internet based activities. This might have led to an underreporting of newer screen time behaviours in 2008 compared with 2001. Acceptable test-retest reliability of the question was found, indicating that the age group had a stable understanding of what screen time activities are and that the question assessed this activity acceptably. Future studies should, however, include detailed questions of screen activities to identify what activities are the main sources of screen time. A further limitation is that weight status was not included in this study. Some results suggest that overweight children spend more time on screen time activities and this relationship could not be assessed in this paper.

Another limitation is that the participating pupils were from two of Norway’s 19 counties only. No large cities are situated within these two counties. However, Oslo is the only large city in Norway (> 250 000 inhabitants), and Norway in general is a rather homogeneous country.

## Conclusion

The present study indicates that there has been a marked reduction in screen time activity outside school in this group of 10–12 year olds from 2001 to 2008 in Norway. During this time period, there has been an increased focus on the benefits of physical activity and the negative health effects of sedentary behavior. This could at least in part explain the observed decrease in screen time activity outside school. The decrease is most evident in 6^th^ graders and in children of parents with college or university level education.

## Competing interest

NCØ and EB declare no competing interests. KIK has since 2006 been employed by the Norwegian Directorate of Health which is responsible for the policy related to physical activity and inactivity.

## Authors’ contributions

KIK conceived the 2001 study. EB conceived the 2008 study. EB and NCØ designed the present study. EB analyzed the data and all authors contributed to the interpretation. NCØ drafted the manuscript, and EB and KIK revised it critically. All authors have approved the final version of the manuscript.

## Pre-publication history

The pre-publication history for this paper can be accessed here:

http://www.biomedcentral.com/1471-2458/13/80/prepub
